# Effects of JFET Region Design and Gate Oxide Thickness on the Static and Dynamic Performance of 650 V SiC Planar Power MOSFETs

**DOI:** 10.3390/ma15175995

**Published:** 2022-08-30

**Authors:** Shengnan Zhu, Tianshi Liu, Junchong Fan, Hema Lata Rao Maddi, Marvin H. White, Anant K. Agarwal

**Affiliations:** Department of Electrical and Computer Engineering, The Ohio State University, Columbus, OH 43210, USA

**Keywords:** SiC power MOSFET, JFET width, JFET doping concentration, gate oxide thickness, orthogonal P^+^ layout, gate-drain capacitance, high-frequency figure-of-merit (HF-FOM)

## Abstract

650 V SiC planar MOSFETs with various JFET widths, JFET doping concentrations, and gate oxide thicknesses were fabricated by a commercial SiC foundry on two six-inch SiC epitaxial wafers. An orthogonal $P^{+}$ layout was used for the 650 V SiC MOSFETs to reduce the ON-resistance. The devices were packaged into open-cavity TO-247 packages for evaluation. Trade-off analysis of the static and dynamic performance of the 650 V SiC power MOSFETs was conducted. The measurement results show that a short JFET region with an enhanced JFET doping concentration reduces specific ON-resistance ($R_{\mathrm{on},\mathrm{sp}}$) and lowers the gate-drain capacitance ($C_{\mathrm{gd}}$). It was experimentally shown that a thinner gate oxide further reduces $R_{\mathrm{on},\mathrm{sp}}$, although with a penalty in terms of increased $C_{\mathrm{gd}}$. A design with 0.5 μm half JFET width, enhanced JFET doping concentration of $5.5\times 10^{16}$ cm^−3^, and thin gate oxide produces an excellent high-frequency figure of merit (HF-FOM) among recently published studies on 650 V SiC devices.

## 1. Introduction

Silicon carbide (SiC) power Metal-Oxide-Semiconductor Field-Effect Transistors (MOSFETs) have been commercialized in a wide range of voltage ratings from 600 V to 1700 V. The launch of 650 V SiC MOSFETs addresses the lower voltage applications, which have traditionally been dominated by Si devices. SiC power MOSFETs outperform Si devices in low switching loss, high switching frequency, low ON-resistance ($R_{\mathrm{on}}$), and high temperature operations [1,2,3]. Hence, designing SiC power MOSFETs with lower $R_{\mathrm{on}}$ and superior switching performance needs to be studied in detail.

JFET region design, including the JFET width and doping concentration, plays a crucial role in optimizing the $R_{\mathrm{on}}$ and switching performance of SiC MOSFETs [4]. Studies of JFET region design for 1 kV and 1.2 kV SiC MOSFETs [5,6] have demonstrated that optimizing JFET width and enhancing the doping concentration of the JFET region can reduce the JFET region resistance and lead to smaller $R_{\mathrm{on}}$ of SiC power MOSFETs. In addition, JFET region design affects the gate-drain capacitance ($C_{\mathrm{gd}}$); $C_{\mathrm{gd}}$ determines the switching performance of 650 V SiC MOSFETs, primarily due to the well-known Miller effect [7]. The product of $C_{\mathrm{gd}}$ and $R_{\mathrm{on}}$ is referred to as the high-frequency figure of merit (HF-FOM) [8]. A lower HF-FOM implies better high-frequency switching performance for devices. Sung and Baliga have reported that a narrow JFET width with a high JFET doping concentration decreases $C_{\mathrm{gd}}$ and improves HF-FOM [9]. The gate-source and the drain-source capacitances, $C_{\mathrm{gs}}$ and $C_{\mathrm{ds}}$, respectively, are affected by JFET width variation through the pitch of the cell, while $C_{\mathrm{gs}}$ and $C_{\mathrm{ds}}$ contribute to the switching loss of SiC power MOSFETs [10].

Gate oxide thickness plays a role in the static and dynamic performance of SiC MOSFETs. As an example, a 27 nm gate oxide was used for 650 V SiC power MOSFETs by Agarwal et al. [11,12], resulting a 1.7× better specific ON-resistance ($R_{\mathrm{on},\mathrm{sp}}$) compared to MOSFETs with a 55 nm gate oxide. Under a certain operation gate voltage, a thinner gate oxide decreases $R_{\mathrm{on},\mathrm{sp}}$ by reducing the channel resistance. However, a thin gate oxide increases the gate oxide capacitance ($C_{\mathrm{ox}}$), and hence increases $C_{\mathrm{gd}}$ and $C_{\mathrm{gs}}$. In addition, a thin gate oxide raises gate oxide reliability issues when sustaining high gate oxide fields [13].

In this work, the authors analyze the performance trade-offs, including threshold voltage ($V_{\mathrm{th}}$), $R_{\mathrm{on},\mathrm{sp}}$, breakdown voltage (BV), and parasitic capacitances for 650 V SiC MOSFETs with different JFET widths, JFET doping concentrations, and gate oxide thicknesses. The 650 V SiC power MOSFETs were fabricated on two six-inch SiC epitaxial wafers by a commercial foundry. The design details and fabrication information are presented in Section 2. The preliminary wafer-level characterizations have been published in [14]. The fabricated devices were packaged for static and dynamic measurements. The experimental methods are explained in Section 3. In Section 4, the experimental results are presented and discussed. Section 5 provides further analysis of the performance trade-offs for the 650 V SiC MOSFETs.

## 2. Device Design and Fabrication

**Figure 1:**
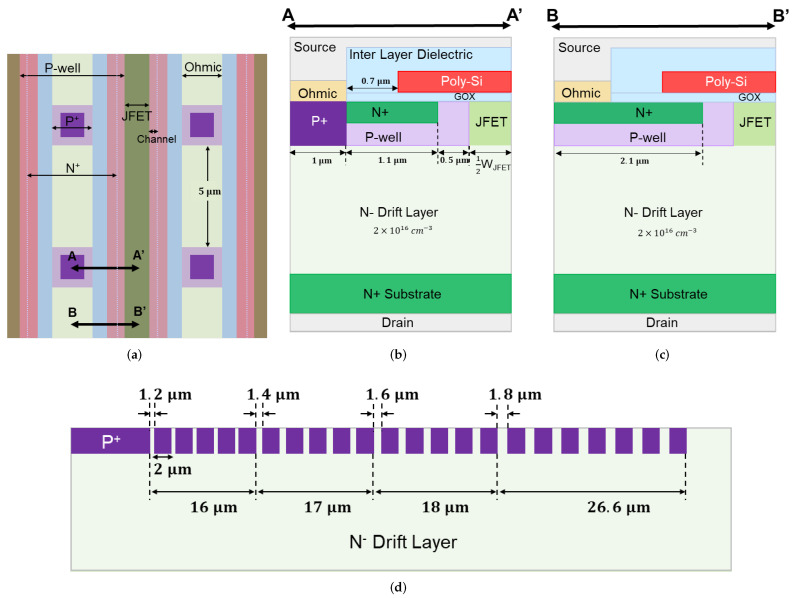
(**a**) Layout design of a SiC power MOSFET with $P^{+}$ located periodically in the center of P-well stripe; (**b**) A-A′ cross-sectional view showing both $P^{+}$ and $N^{+}$; (**c**) B-B′ cross-sectional view showing extended $N^{+}$ source; (**d**) cross-sectional view of the edge termination of the fabricated 650V SiC power MOSFETs.

The layout design of a 650 V SiC MOSFET is shown in Figure 1a. The layout is in a stripe pattern, with square $P^{+}$ regions located periodically in the center of the P-well stripe. The orthogonal $P^{+}$ layout reduces the $R_{\mathrm{on}}$ of the MOSFETs by reducing the cell pitch compared to the traditional linear striped $P^{+}$ layout. The cross-section along the A-A${}^{\prime }$ cutline is shown in Figure 1b. The half-cell pitch consists of $P^{+}$ width (1 μm), $N^{+}$ source width (1.1 μm), channel length (0.5 μm), and half JFET width ($\frac{1}{2}W_{\mathrm{JFET}}$). The spacing between the source contact and polysilicon gate is 0.7 μm. The ohmic contact width is 1 μm. The cross-section along B-B′ (Figure 1c) shows the layout with only $N^{+}$ source. The extended $N^{+}$ source replaces the $P^{+}$ in the A-A${}^{\prime }$ half-cell pitch and produces a total $N^{+}$ source width of 2.1 μm. Four devices with different half-JFET widths were designed ($\frac{1}{2}W_{\mathrm{JFET}}$ = 0.4, 0.5, 0.6, and 0.75 μm).

Twenty-two $P^{+}$ guard rings were used as the edge termination for all layouts. Each guard ring had a width of 2 μm. A cross-sectional view of the edge termination is shown in Figure 1d. The edge termination can be divided into four sections. The spacing for each section is illustrated in Figure 1d; spacing was identical in each section. The total length of the edge termination was 77.6 μm.

Different JFET doping concentrations ($N_{\mathrm{JFET}}$) and gate oxide thicknesses ($t_{\mathrm{ox}}$) were utilized during the fabrication of the devices. The devices were fabricated on two six-inch 4H-SiC wafers (wafer 1 and wafer 2) with n-type epitaxial layers on $N^{+}$ substrates. The substrates were thinned to reduce the resistance. The epitaxial layer was doped with nitrogen with a doping concentration of $2\times 10^{16}$ cm^−3^. Ion implantation of nitrogen was used to form the JFET region and $N^{+}$ source; $N_{\mathrm{JFET}}$ = $4\times 10^{16}$ cm^−3^ and $N_{\mathrm{JFET}}$ = $5.5\times 10^{16}$ cm^−3^ were used for wafers 1 and 2, respectively. Aluminum ions were implanted to form the P-well and $P^{+}$ region. The gate oxide was grown after the implantation and activation annealing processes. The gate oxide thicknesses on wafers 1 and 2 are represented as $t_{\mathrm{ox}1}$ (36∼44 nm) and $t_{\mathrm{ox}2}$ (32∼38 nm), respectively; $t_{\mathrm{ox}2}$ is 12.5% less than $t_{\mathrm{ox}1}$. Details of the gate oxide thicknesses have been discussed previously in [14]. Self-alignment technology was utilized to form the MOS channel. Fabrication was completed following the standard process flow of commercial SiC MOSFETs.

**Figure 2:**
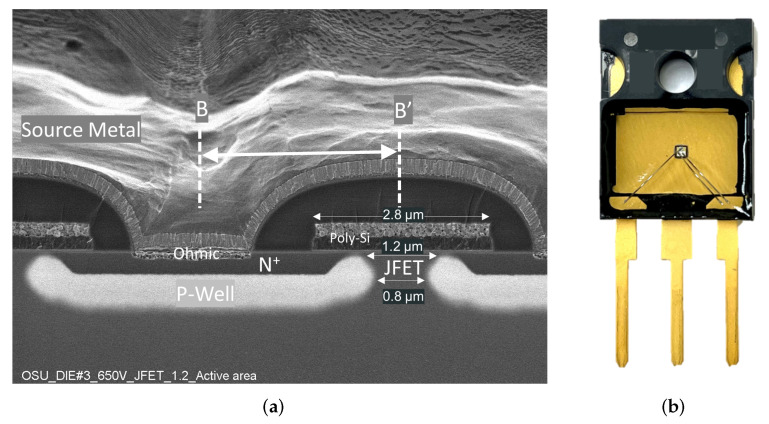
(**a**) Cross-sectional SEM image along B-B^′^ of the fabricated 650V SiC power MOSFETs with $\frac{1}{2}W_{\mathrm{JFET}}$ = 0.6 μm and (**b**) 650V SiC power MOSFET in a open-cavity TO-247 package.

The design parameters and experimental results for all devices are summarized in Table 1 (Section 5). Figure 2a shows a cross-sectional SEM image (along BB’ in Figure 1c) of the fabricated 650 V SiC MOSFET ($\frac{1}{2}W_{\mathrm{JFET}}$ = 0.6 μm) on wafer 1. Due to the lateral straggle of Aluminum implantation in the P-well, the narrowest portion of the JFET region is reduced by 0.2 μm on each side.

## 3. Experimental Methods

### 3.1. Device Packaging

The fabricated MOSFETs were diced and packaged into open cavity TO-247 packages, as shown in Figure 2b. A single 5-mil aluminum wire bond was used for the gate terminal, while two-wire bonds were attached on the the source area to decrease the parasitic resistance. Silicone dielectric gel was used to fill the cavity to protect the bare die. Five copies of each layout design on wafers 1 and 2 were packaged.

### 3.2. Device Characterization

The static performance of the MOSFETs, including the transfer, output, and blocking characteristics, were measured with a Keysight B1506A semiconductor parameter analyzer. We extracted $V_{\mathrm{th}}$ at a drain current of 1 mA from the transfer characteristics tested under a drain bias of 100 mV. The output characteristics were measured under a gate bias of 20 V, with the drain voltage swept from 0 to 2 V. We obtained the $R_{\mathrm{on}}$ of the device under test (DUT) at a drain bias of 1.5 V; BV was obtained from the blocking I-V characteristics at a current of 100 μA, while $C_{\mathrm{gd}}$, $C_{\mathrm{gs}}$, and $C_{\mathrm{ds}}$ were measured up to a drain bias of 400 V at a frequency of 100 kHz using a Keysight B1505A semiconductor parameter analyzer.

## 4. Device Characteristics and Discussion

The measured device characteristics for the packaged 650 V SiC MOSFETs with different designs are illustrated and compared in this section.

### 4.1. Threshold Voltage

**Figure 3:**
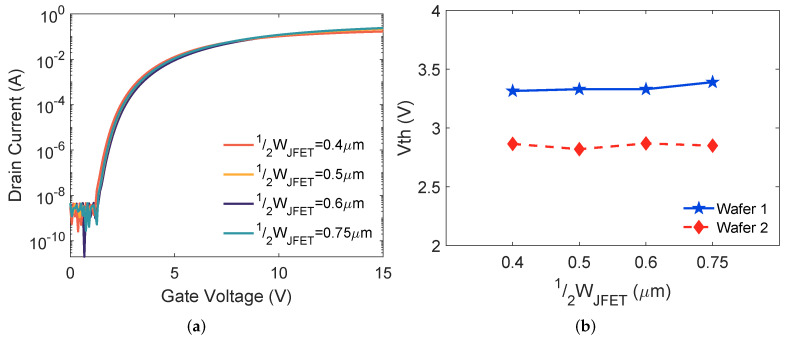
(**a**) Measured transfer characteristics of the packaged 650V SiC MOSFETs on wafer 1 and (**b**) $V_{\mathrm{th}}$ variation as a function of $\frac{1}{2}W_{\mathrm{JFET}}$ for MOSFETs on wafers 1 and 2.

The transfer characteristics for the devices with different $\frac{1}{2}W_{\mathrm{JFET}}$ on wafer 1 are plotted in Figure 3a. Typical transfer curves of SiC MOSFETs were obtained from all the DUTs. The average $V_{\mathrm{th}}$ from the five copies of each design is plotted in Figure 3b. Minimal $V_{\mathrm{th}}$ variation was observed for wafers 1 and 2 when increasing $\frac{1}{2}W_{\mathrm{JFET}}$.

The $V_{\mathrm{th}}$ of the MOSFETs on wafer 2 is ∼0.5 V smaller than wafer 1, as shown in Figure 3b. The thinner gate oxide contributes to the $V_{\mathrm{th}}$ reduction; here, $V_{\mathrm{th}}$ is defined as [8]:

(1) $$V_{\mathrm{th}}=\Phi_{\mathrm{MS}}+\frac{\sqrt{4\varepsilon_{\mathrm{SiC}}kTN_{A}\mathrm{In}\left(N_{A}/n_{i}\right)}-Q_{\mathrm{ox}}}{C_{\mathrm{ox}}}+\frac{2kT}{q}\ln\left(\frac{N_{A}}{n_{i}}\right),$$

where $\Phi_{\mathrm{MS}}$ is the metal–semiconductor work function difference, $\varepsilon {}_{\mathrm{ox}}$ is the permittivity of SiC, *k* and *T* are the Boltzmann constant and temperature, respectively, $n_{i}$ is the intrinsic carrier concentration of SiC, *q* is the electric charge, $Q_{\mathrm{ox}}$ is the total effective charge in the oxide (the sum of the fixed and interface charges), and $N_{A}$ is the net p-type doping concentration at the channel region; $C_{\mathrm{ox}}$ is the gate oxide capacitance, which is given as

(2) $$C_{\mathrm{ox}}=\frac{\varepsilon_{\mathrm{ox}}}{t_{\mathrm{ox}}}$$

where $\varepsilon_{\mathrm{ox}}$ is the permittivity of oxide. Comparing wafer 2 to wafer 1, higher $N_{\mathrm{JFET}}$ reduces $N_{A}$ by the effect of the counter doping at the surface. Additionally, the thinner gate oxide of wafer 2 increases $C_{\mathrm{ox}}$. According to (1), the reduced $N_{A}$ at the surface and increased $C_{\mathrm{ox}}$ lead to smaller $V_{\mathrm{th}}$ of the MOSFETs on wafer 2.

### 4.2. Specific ON-Resistance

**Figure 4:**
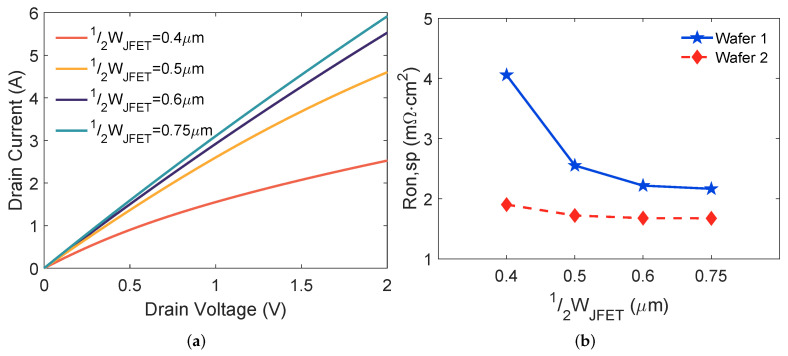
(**a**) Measured output characteristics of the SiC MOSFETs on wafer 1 and (**b**) $R_{\mathrm{on},\mathrm{sp}}$ variation as a function of $\frac{1}{2}W_{\mathrm{JFET}}$ for MOSFETs on wafers 1 and 2.

Figure 4a shows the output characteristics at a gate bias of 20 V for devices on wafer 1. Drain current increases with a wider JFET region. Figure 4b plots $R_{\mathrm{on},\mathrm{sp}}$ versus $\frac{1}{2}W_{\mathrm{JFET}}$ variation. For both wafers 1 and 2, $R_{\mathrm{on},\mathrm{sp}}$ is reduced when increasing $\frac{1}{2}W_{\mathrm{JFET}}$ because a larger $\frac{1}{2}W_{\mathrm{JFET}}$ provides lower JFET region resistance [14]. With the same $\frac{1}{2}W_{\mathrm{JFET}}$, $R_{\mathrm{on},\mathrm{sp}}$ reduction from wafer 1 to wafer 2 is contributed by thinner gate oxide and higher $N_{\mathrm{JFET}}$. A considerable (1.6×) $R_{\mathrm{on},\mathrm{sp}}$ reduction is observed when $\frac{1}{2}W_{\mathrm{JFET}}$ rises from 0.4 μm to 0.5 μm on wafer 1, while the tendency is weaker for wafer 2. These results indicate that thinner gate oxide and higher $N_{\mathrm{JFET}}$ make $R_{\mathrm{on},\mathrm{sp}}$ less susceptible to $\frac{1}{2}W_{\mathrm{JFET}}$ variation.

### 4.3. Breakdown Voltage

**Figure 5:**
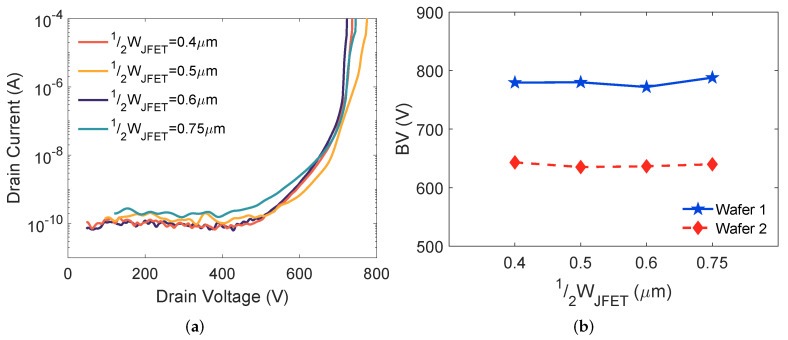
(**a**) Measured blocking characteristics of the SiC MOSFETs on wafer 1 and (**b**) Maximum BV as a function of $\frac{1}{2}W_{\mathrm{JFET}}$ for MOSFETs on wafers 1 and 2.

The blocking characteristics for MOSFETs on wafer 1 are shown in Figure 5a. All DUTs maintain low leakage currents (∼100 pA) under drain voltage up to 550 V. The drain to source breakdown of a planar SiC MOSFET is triggered by avalanche breakdown, and both $N_{\mathrm{JFET}}$ and $\frac{1}{2}W_{\mathrm{JFET}}$ have little effect on the BV determined by avalanche breakdown [5]. Our experimental results (Figure 5b) show that the BV of 650 V SiC MOSFETs is minimally changed by $\frac{1}{2}W_{\mathrm{JFET}}$ variation.

A maximum BV of about 780 V is achieved for devices on wafer 1. The BV for MOSFETs on wafer 2 is ∼640 V. The 18% BV drop from wafer 1 to wafer 2 is mainly caused by the difference in drift layer doping. The drift layer doping concentrations can be extracted from the C-V measurement of MOS capacitors on both wafers [15]. The extracted drift layer doping concentrations are $1.8\times 10^{16}$ cm^−3^ and $2.1\times 10^{16}$ cm^−3^ for wafers 1 and 2, respectively. This difference explains the reduction of BV on wafer 2.

Although BV does not change with $\frac{1}{2}W_{\mathrm{JFET}}$ variation, a smaller $\frac{1}{2}W_{\mathrm{JFET}}$ improves the gate oxide reliability of the MOSFETs by better shielding the gate oxide on the top of the JFET region from high oxide fields under the blocking condition [14,16]. These high oxide fields may cause high gate leakage currents, degrade the gate oxide, and reduce the oxide lifetime [13,17], and can lead to failures during High-Temperature Reverse Bias (HTRB) testing.

### 4.4. Device Capacitances

**Figure 6:**
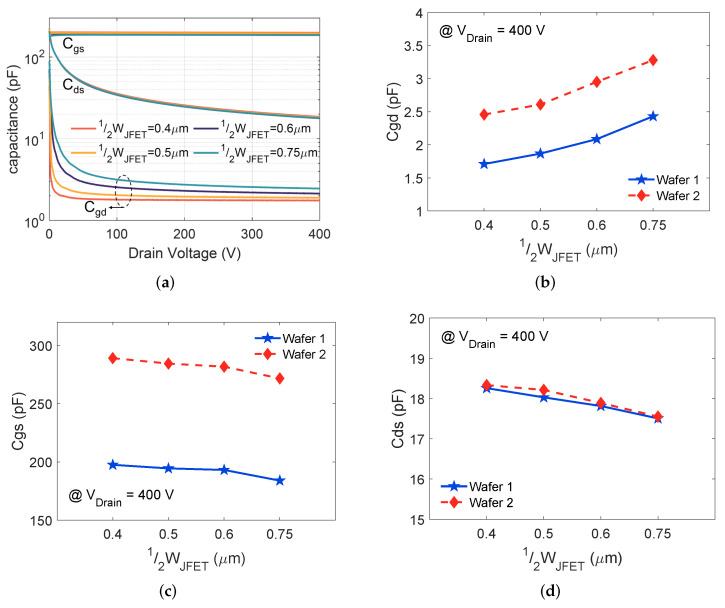
(**a**) Measured device capacitances vs. drain voltage at 100 kHz of the SiC MOSFETs on wafer 1, (**b**) $C_{\mathrm{gd}}$, (**c**) $C_{\mathrm{ds}}$, and (**d**) $C_{\mathrm{gs}}$ variation as a function of $\frac{1}{2}W_{\mathrm{JFET}}$ for MOSFETs on wafers 1 and 2.

The device capacitances as a function of the applied drain bias for 650 V MOSFETs on wafer 1 are shown in Figure 6a. As expected, the measured $C_{\mathrm{gd}}$ and $C_{\mathrm{ds}}$ are nonlinear functions of the drain bias, while $C_{\mathrm{gs}}$ stays relatively constant with increasing drain bias. The extracted $C_{\mathrm{gd}}$, $C_{\mathrm{ds}}$, and $C_{\mathrm{gs}}$ as function of $\frac{1}{2}W_{\mathrm{JFET}}$ for the MOSFETs on wafers 1 and 2 are shown in Figure 6b–d, respectively.

**Figure 7:**
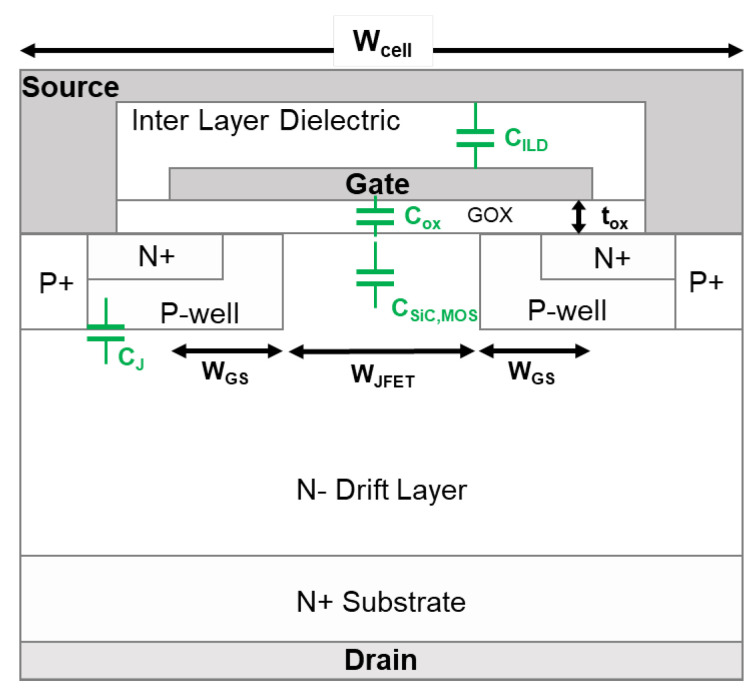
Device capacitance components.

When extending $\frac{1}{2}W_{\mathrm{JFET}}$, $C_{\mathrm{gd}}$ increases. For a planar SiC MOSFET, $C_{\mathrm{gd}}$ is formed by the overlap between the gate and drain electrodes. A complete cell cross-section in Figure 7, illustrating the various device capacitance components. Here, $C_{\mathrm{gd}}$ is composed of $C_{\mathrm{ox}}$ and depletion region capacitance ($C_{\mathrm{SiC},\mathrm{MOS}}$) under the gate oxide; $C_{\mathrm{gd}}$ is defined as follows:

(3) $$C_{\mathrm{gd}}=\frac{W_{\mathrm{JFET}}}{W_{\mathrm{cell}}}\left(\frac{C_{\mathrm{ox}}\;\cdot \;C_{\mathrm{SiC},\mathrm{MOS}}}{C_{\mathrm{ox}}+C_{\mathrm{SiC},\mathrm{MOS}}}\right)\;\cdot \;A_{\mathrm{active}}.$$

Equation (3) is based on [8], where $W_{\mathrm{cell}}$ refers to the cell pitch, $A_{\mathrm{active}}$ represents the active area of the device, $C_{\mathrm{ox}}$ stays constant for the devices with the same $t_{\mathrm{ox}}$, and $C_{\mathrm{SiC},\mathrm{MOS}}$ is determined by the depletion layer thickness under the gate oxide, which does not change for devices with the same $N_{\mathrm{JFET}}$ and which sustain a specific drain bias. According to (3), $C_{\mathrm{gd}}$ increases when increasing $W_{\mathrm{JFET}}$, which agrees with the measured results for both wafers 1 and 2 in Figure 6b.

Comparing wafer 2 to wafer 1, $t_{\mathrm{ox}}$ drops by 12.5%. Correspondingly, $C_{\mathrm{ox}}$ increases by 14.3% and leads to $C_{\mathrm{gd}}$ increasing. The enhanced $N_{\mathrm{JFET}}$ of wafer 2 affects $C_{\mathrm{SiC},\mathrm{MOS}}$ by changing the thickness and the width of the depletion layer [7,18]. It is challenging to identify the change of $C_{\mathrm{SiC},\mathrm{MOS}}$ quantitatively, as the depletion layer varies with the gate-drain bias, p-well potential, and doping concentration of the JFET and drift layer [19]. The results in [9] demonstrate that a higher $N_{\mathrm{JFET}}$ leads to a higher $C_{\mathrm{gd}}$. Thus, the overall outcome from lowering $t_{\mathrm{ox}}$ and increasing $N_{\mathrm{JFET}}$ is the increase of $C_{\mathrm{gd}}$. The measured $C_{\mathrm{gd}}$ for MOSFETs on wafer 2 is about 1.4× higher than those of wafer 1 under a given $\frac{1}{2}W_{\mathrm{JFET}}$, as shown in Figure 6b.

Note that $C_{\mathrm{gs}}$ consists of the overlap capacitance of the gate electrode with source plus channel region and the parallel capacitance across the gate and source metallization ($C_{\mathrm{ILD}}$) [20]; $C_{\mathrm{gs}}$ in the active area of a 650 V SiC MOSFET is addressed as

(4) $$C_{\mathrm{gs}}=\left(\frac{2W_{\mathrm{GS}}}{W_{\mathrm{cell}}}C_{\mathrm{ox}}+\frac{2W_{\mathrm{GS}}+W_{\mathrm{JFET}}}{W_{\mathrm{cell}}}C_{\mathrm{ILD}}\right)\;\cdot \;A_{\mathrm{active}},$$

where $W_{\mathrm{GS}}$ is the total length of the overlap between gate and $N^{+}$ source and the channel region and $C_{\mathrm{ILD}}$ is the inter-layer dielectric capacitance, which stays constant for all the devices due to the same fabrication process being used for wafers 1 and 2.

Among the designs on the same wafer, increasing $W_{\mathrm{JFET}}$ reduces the coefficients of $C_{\mathrm{ox}}$ and $C_{\mathrm{ILD}}$ in (4) and leads to the increase of $C_{\mathrm{gs}}$. The measured $C_{\mathrm{gs}}$ verifies the variation for both wafer 1 and wafer 2 in Figure 6c. For a specific $W_{\mathrm{JFET}}$, a thinner gate oxide increases $C_{\mathrm{ox}}$, and hence result in a higher $C_{\mathrm{gs}}$ according to (4). This explains the higher $C_{\mathrm{gs}}$ measured on wafer 2 compared to $C_{\mathrm{gs}}$ on wafer 1.

As $C_{\mathrm{ds}}$ is driven by the depletion layer formation at the P-well and drift region interface, the total $C_{\mathrm{ds}}$ in the active area of a 650 V SiC MOSFET is expressed as

(5) $$C_{\mathrm{ds}}=\frac{W_{\mathrm{cell}}-W_{\mathrm{JFET}}}{W_{\mathrm{cell}}}C_{J}\;\cdot \;A_{\mathrm{active}},$$

where $C_{J}$ is the junction capacitance per unit area, which is determined by the depletion layer thickness. All the DUTs in this work have similar doping concentration of the epi-layer. The bottom of the P-well is heavily doped, meaning that the depletion thickness in the p-well region can be neglected. Thus, under a particular drain bias, the depletion layer thickness stays almost the same for all DUTs, which results in similar $C_{J}$. According to (5), increasing $W_{\mathrm{JFET}}$ reduces $C_{\mathrm{ds}}$, corresponding to the measured results in Figure 6d for both wafer 1 and wafer 2. In addition, the measured $C_{\mathrm{ds}}$ under a certain $\frac{1}{2}W_{\mathrm{JFET}}$ is almost the same for the MOSFETs on both wafers, which is due to the fact that $t_{\mathrm{ox}}$ and $N_{\mathrm{JFET}}$ are not involved in (5).

## 5. Trade-Offs

Table 1 summarizes the design information and experimental results for the 650 V SiC MOSFETs. HF-FOM is included to evaluate the performance of the devices.

The variation of $\frac{1}{2}W_{\mathrm{JFET}}$ influences $R_{\mathrm{on},\mathrm{sp}}$ and the device capacitance. When reducing the $\frac{1}{2}W_{\mathrm{JFET}}$ from 0.75 μm to 0.4 μm, (1) $R_{\mathrm{on},\mathrm{sp}}$ increases by 1.9× for wafer 1 and 1.1× for wafer 2; (2) $C_{\mathrm{gd}}$ decreases by 1.4× for wafer 1 and 1.3× for wafer 2; and (3) less than 7% and 4% increase are identified for $C_{\mathrm{gs}}$ and $C_{\mathrm{ds}}$, respectively.

Comparing the performance of the MOSFETs on wafer 2 to those on wafer 1, higher $N_{\mathrm{JFET}}$ and thinner gate oxide have the following benefits: (1) $R_{\mathrm{on},\mathrm{sp}}$ is further reduced and the variation of $R_{\mathrm{on},\mathrm{sp}}$ caused by variation in $\frac{1}{2}W_{\mathrm{JFET}}$ is mitigated; (2) $V_{\mathrm{th}}$ is reduced by about 10%; and (3) a low HF-FOM of 699 mΩ·pF is obtained at $\frac{1}{2}W_{\mathrm{JFET}}$ of 0.5 μm. The trade-offs are that $C_{\mathrm{gs}}$ and $C_{\mathrm{gd}}$ are increased and the oxide field on the top of the JFET region may rise; $C_{\mathrm{ds}}$ is not affected. BV should not be affected either, assuming that the drift layer doping and thickness remain the same.

Combining the above analysis, a narrower JFET region with a thinner gate oxide and enhanced $N_{\mathrm{JFET}}$ produce optimized designs for 650 V SiC MOSFETs. A small $W_{\mathrm{JFET}}$ reduces $C_{\mathrm{gd}}$. The increased $R_{\mathrm{on},\mathrm{sp}}$ thanks to smaller $W_{\mathrm{JFET}}$ can be compensated for by thinner gate oxide and higher $N_{\mathrm{JFET}}$. A narrow JFET region helps to shield the gate oxide on the top of JFET region from high oxide fields that may be induced by the thin gate oxide and high $N_{\mathrm{JFET}}$.

**Table 1:** Summary of design information and experimental results.

	Design Information					Experimental Results												
	$\frac{1}{2}W_{\mathrm{JFET}}$ [ μm]	$t_{\mathrm{ox}}$ [nm]	N_JFET_ [cm^−3^]	Cell Pitch [ μm]	Active Area [mm ${}^{2}$ ]	$R_{\mathrm{on},\mathrm{sp}}$ [m Ω·cm^2^ ]	$R_{\mathrm{on},\mathrm{sp}}$ Std.	V_th_ [V]	V_th_ Std.	BV [V]	BV Std.	C_gs_ [pF]	C_gs_ Std.	C_ds_ [pF]	C_ds_ Std.	C_gd_ [pF]	C_gd_ Std.	HF-FOM (C_gd_ × $R_{\mathrm{on}}$) [mΩ·pF]
Wafer 1	0.4	$t_{\mathrm{ox}1}$ *	$4\times 10^{16}$	6	0.64	4.06	0.77	3.3	0.08	780	20.5	197	4.0	18.3	0.2	1.7	0.05	1078
	0.5			6.2	0.64	2.55	0.07	3.3	0.09	780	19.1	194	2.6	18.0	0.3	1.9	0.05	757
	0.6			6.4	0.64	2.22	0.03	3.3	0.08	772	19.9	193	2.9	17.8	0.2	2.1	0.03	728
	0.75			6.7	0.64	2.16	0.04	3.4	0.10	788	21.7	184	3.0	17.5	0.3	2.4	0.03	810
Wafer 2	0.4	$t_{\mathrm{ox}2}$ *	$5.5\times 10^{16}$	6	0.64	1.90	0.08	2.9	0.11	643	31.6	289	1.5	18.3	0.3	2.5	0.05	742
	0.5			6.2	0.64	1.72	0.03	2.8	0.10	635	36.8	284	2.5	18.2	0.3	2.6	0.05	699
	0.6			6.4	0.64	1.68	0.03	2.9	0.09	637	30.7	282	1.8	17.9	0.4	3.0	0.05	788
	0.75			6.7	0.64	1.67	0.03	2.9	0.11	640	45.0	272	2.0	17.6	0.3	3.3	0.03	861

* $t_{\mathrm{ox}2}$ is 12.5% less than $t_{\mathrm{ox}1}$.

## 6. Conclusions

In this paper, 650 V SiC MOSFETs were designed, fabricated, packaged, and characterized. The on-state and dynamic performance trade-offs due to the JFET region and gate oxide thickness design were then analyzed. Our experimental results show that a narrow JFET width and enhanced JFET doping concentration lead to low $R_{\mathrm{on},\mathrm{sp}}$, low $C_{\mathrm{gd}}$, low HF-FOM, and better gate oxide reliability without degrading the $V_{\mathrm{th}}$ and BV. The increases in $C_{\mathrm{gs}}$ and $C_{\mathrm{ds}}$ with reduction in JFET width are relatively small in comparison with the reduction of $C_{\mathrm{gd}}$. In addition, we have shown that $R_{\mathrm{on},\mathrm{sp}}$ can be further reduced with a thinner gate oxide, although this incurs a penalty in terms of increased $C_{\mathrm{gd}}$. 

## Data Availability

Not applicable.

## Abbreviations

The following abbreviations are used in this manuscript:

SiC	Sillicon Carbide
MOSFET	Metal–Oxide Semiconductor Field-Effect Transistor
JFET	Junction Field Effect Transistor
HF-FOM	High-Frequency Figure Of Merit
DUT	Device Under Test
